# Serum Zinc and Selenium Are Independently Associated with Acute-Phase Inflammatory Markers in Critically Ill Patients: A Retrospective Observational Study

**DOI:** 10.3390/nu18152508

**Published:** 2026-08-03

**Authors:** Hyoseok Oh, Seung Min Baik, Jae-Myeong Lee

**Affiliations:** 1 Division of Acute Care Surgery, Department of Surgery, Korea University Anam Hospital, Seoul 02841, Republic of Korea; 2 Division of Critical Care Medicine, Department of Surgery, Ewha Womans University Mokdong Hospital, Seoul 07985, Republic of Korea

**Keywords:** zinc, selenium, copper, manganese, trace elements, C-reactive protein, procalcitonin, critical illness, intensive care unit, inflammation

## Abstract

**Background/Objectives:** Zinc, selenium, copper, and manganese are essential cofactors for immune defense and redox homeostasis, yet their independent associations with routinely measured inflammatory markers in critically ill patients remain incompletely characterized. We aimed to examine these associations in intensive care unit (ICU) patients. **Methods:** This retrospective observational study enrolled 267 adult ICU patients at a single tertiary center. Serum trace element levels were measured at ICU admission and serially in a subset. Inflammatory markers—white blood cell count, neutrophil percentage, C-reactive protein (CRP), and procalcitonin—were measured concurrently. Hierarchical regression was performed at three adjustment levels, with Model 2 further adjusted for all remaining trace elements. A supplementary serial-level analysis used per-patient median concentrations. **Results:** In the fully adjusted model, zinc was independently and negatively associated with CRP (β = −0.68; 95% CI, −1.11 to −0.25; *p* = 0.002) and procalcitonin (exp[β] = 0.973; 95% CI, 0.961–0.986; *p* < 0.001), and selenium with CRP (β = −0.87; 95% CI, −1.48 to −0.25; *p* = 0.006). Copper showed a positive association with CRP exclusively in the fully adjusted model (β = 0.60; *p* = 0.006), consistent with ceruloplasmin’s role as a positive acute-phase reactant. Serial-level analyses corroborated these findings. No trace element was associated with neutrophil percentage. **Conclusions:** Lower serum zinc and selenium were independently associated with higher acute-phase inflammatory markers in critically ill ICU patients. These findings highlight the potential clinical relevance of trace element assessment in critically ill patients. Prospective studies are needed to determine whether targeted repletion can modify inflammation or improve clinical outcomes.

## 1. Introduction

Critically ill patients admitted to the intensive care unit (ICU) are characterized by a state of profound systemic inflammation, driven by dysregulated immune activation and organ dysfunction in response to insults including sepsis, trauma, and major surgery [1]. Within this context, trace elements—zinc, selenium, copper, and manganese—have attracted considerable interest, as they serve as essential cofactors for enzymes central to immune defense and redox homeostasis, including superoxide dismutase, glutathione peroxidase, and catalase [2,3].

Serum concentrations of trace elements fall markedly during critical illness. Zinc and selenium deficiencies are particularly prevalent, documented in up to 75% and 36% of ICU patients at admission, respectively [4]. These decrements arise through multiple mechanisms: redistribution driven by inflammatory cytokines, increased urinary losses, reduced synthesis of carrier proteins such as albumin, and intrahepatic sequestration mediated by metallothionein induction [5]. The resulting hypozincemia and hyposelenemia are not merely epiphenomena; experimental evidence indicates they actively amplify the inflammatory cascade. Zinc deficiency augments NF-κB signaling, resulting in exaggerated production of TNF-α, IL-1β, and IL-6 [6,7], while selenium is required for the biosynthesis of selenoproteins that modulate cytokine expression and attenuate oxidative injury [8].

Copper behaves differently from zinc and selenium during the acute-phase response. As the principal carrier of copper in plasma, ceruloplasmin is a positive acute-phase reactant whose synthesis increases in parallel with inflammatory stimulation [2,3]. Consequently, serum copper levels may rise—rather than fall—in the setting of critical illness, a pattern that complicates the interpretation of copper status in this population [4,9].

Clinical inflammatory markers, including C-reactive protein (CRP) and procalcitonin, are routinely used in the ICU to assess infection severity, guide antibiotic therapy, and monitor treatment response [10]. Despite the biological plausibility of a connection between trace element status and inflammatory marker levels, most clinical studies have examined trace elements primarily in relation to organ failure scores, ICU length of stay, or mortality [4,11,12,13]. The specific associations between individual trace element concentrations and routinely measured inflammatory markers—assessed both cross-sectionally at admission and longitudinally across the ICU stay—remain less clearly characterized.

We therefore conducted a retrospective study of ICU patients in whom zinc, selenium, copper, and manganese were measured alongside concurrent inflammatory markers. We examined bivariate correlations and performed hierarchical multivariable regression analyses to identify independent associations, adjusting for key clinical confounders. To assess whether admission-level findings reflected sustained relationships throughout the ICU stay, a supplementary serial analysis using per-patient median trace element concentrations was also performed.

## 2. Methods

### 2.1. Study Design and Population

This retrospective observational study was conducted at Korea University Anam Hospital, Seoul, South Korea. Adult patients (≥18 years) admitted to the intensive care unit (ICU) between 1 December 2016 and 31 December 2021 were screened. Patients were eligible for inclusion if they underwent serum trace element testing for at least one of zinc, selenium, copper, or manganese as part of routine clinical care and had concurrently measured inflammatory markers, including white blood cell (WBC) count, C-reactive protein (CRP), and procalcitonin. Trace element testing was not performed uniformly in all ICU patients but was ordered at the discretion of the treating physician based on clinical indication, such as prolonged ICU stay, refractory infection, suspected nutritional deficiency, or failure to wean from mechanical ventilation.

Patients were excluded if trace element results were unavailable, if concurrent inflammatory markers were not measured, or if they had hematologic malignancy, given the potential disease-specific confounding effects on trace element metabolism and inflammatory markers. For patients with repeated ICU admissions during the study period, only the first ICU admission was included. APACHE II score was collected as a measure of disease severity and was not used as an inclusion criterion. A total of 267 patients were included in the final analysis.

The study was approved by the Institutional Review Board of Korea University Anam Hospital (IRB No. 2026AN0325; approved on 19 June 2026), and the requirement for informed consent was waived due to the retrospective nature of the study.

### 2.2. Data Collection

Demographic and clinical data were collected from electronic medical records, including age, sex, Acute Physiology and Chronic Health Evaluation (APACHE) II score, serum albumin, serum creatinine, use of continuous renal replacement therapy (CRRT), and in-hospital mortality.

Serum trace element levels—zinc, selenium, copper, and manganese—were measured at ICU admission and, in a subset of patients, at additional time points during the ICU stay based on clinical indication. Iron was excluded from the analysis due to a high proportion of missing values (260/267, 97.4%). Inflammatory and immune markers, including white blood cell (WBC) count, neutrophil percentage, C-reactive protein (CRP), and procalcitonin, were measured concurrently at each assessment.

### 2.3. Statistical Analysis

Baseline characteristics were summarized using descriptive statistics. Continuous variables were expressed as median with interquartile range (IQR), and categorical variables as frequencies and percentages.

Initial-level analysis. Bivariate associations between trace element levels measured at ICU admission and concurrently measured inflammatory markers were assessed using Pearson correlation coefficients (r) and Spearman rank correlation coefficients (ρ). To examine independent associations, a hierarchical regression strategy with three adjustment levels was applied: (1) crude model (univariate); (2) Model 1, adjusted for sex, age, APACHE II score, albumin, creatinine, and CRRT; and (3) Model 2, further adjusted for the remaining three trace elements. The skewness values of WBC count, neutrophil percentage, CRP, and procalcitonin were 1.81, −2.31, 0.70, and 2.65, respectively. For outcomes exhibiting substantial positive (right) skewness exceeding 2 (procalcitonin), generalized linear models (GLM) with a Gamma distribution and log-link function were used; results are expressed as exp(β), representing the multiplicative change in the expected outcome per unit increase in the trace element. The remaining outcomes were analyzed using ordinary least squares (OLS) regression, with results expressed as unstandardized coefficients (β) with 95% confidence intervals (CIs). Although neutrophil percentage showed negative skewness, a Gamma distribution was not applicable given its left-skewed distribution, and OLS regression was retained.

Because copper and manganese were measured in a smaller subset of patients, inclusion of all four trace elements in Model 2 reduced the analyzable sample size. Therefore, we performed an additional sensitivity analysis (Model 2a) in which zinc and selenium were mutually adjusted for one another together with the same clinical covariates used in Model 1, but without adjustment for copper or manganese.

Serial-level analysis. As a supplementary analysis to evaluate whether the associations observed at admission were consistent with trace element levels sustained throughout the ICU stay, we derived a per-patient summary exposure by computing the median of all available serial measurements for each trace element. Given that serial measurements were obtained at the discretion of the treating clinician rather than at predefined protocol time points, the median was chosen as a robust summary measure of each patient’s overall trace element status during the ICU stay. The same hierarchical regression framework and model selection criteria were applied to assess associations between these serial median exposures and the corresponding inflammatory markers. Because no trace element showed a significant association with neutrophil percentage in the initial-level analysis, serial-level results for this outcome were considered exploratory and are reported in the Supplementary Material.

Cases with missing values in any variable included in a given model were handled by complete-case analysis, and sample sizes for each model are reported in the corresponding tables. All statistical analyses were performed using Python 3.9 with the statsmodels (v0.14) and SciPy (v1.10) packages. A two-sided *p*-value < 0.05 was considered statistically significant.

## 3. Results

### 3.1. Baseline Characteristics

The baseline characteristics of the 267 patients are summarized in Table 1. The median age was 72.0 years (IQR, 60.5–80.0), and 170 patients (63.7%) were male. The median APACHE II score was 29.0 (IQR, 20.0–35.0), reflecting a high-acuity population. Forty-nine patients (18.4%) received CRRT, and in-hospital mortality was 44.2% (118/267).

Median serum trace element levels at admission were: zinc, 49.0 µg/dL (IQR, 37.0–66.0); selenium, 54.0 µg/L (IQR, 44.0–66.8); copper, 75.0 µg/dL (IQR, 58.2–94.8); and manganese, 1.3 µg/L (IQR, 0.9–1.9). Copper and manganese had substantial missing data (39.3% and 39.7%, respectively). Median inflammatory marker values were: WBC, 11.3 × 10^3^/µL (IQR, 7.3–15.4); neutrophil percentage, 86.1% (IQR, 78.9–90.6); CRP, 126.3 mg/L (IQR, 54.4–181.1); and procalcitonin, 2.3 ng/mL (IQR, 0.6–10.7).

### 3.2. Initial-Level Analysis

**Figure 1:**
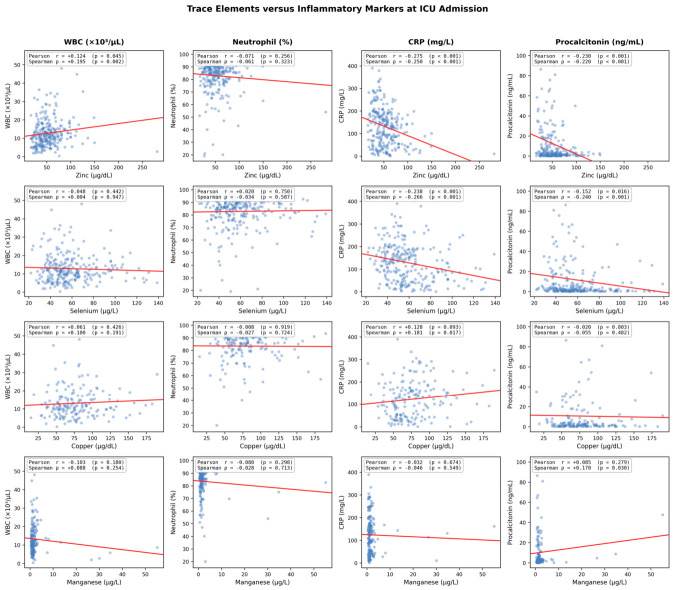
Scatter plots of bivariate associations between trace element levels and inflammatory markers at ICU admission. Each panel displays a single trace element (*x*-axis) plotted against an inflammatory marker (*y*-axis), with a linear regression trend line (red). Rows represent trace elements—zinc (µg/dL), selenium (µg/L), copper (µg/dL), and manganese (µg/L), from top to bottom—and columns represent inflammatory markers—white blood cell count (×10^3^/µL), neutrophil percentage (%), C-reactive protein (mg/L), and procalcitonin (ng/mL), from left to right. Axis ranges are held constant within each row for the trace element and within each column for the inflammatory marker to permit direct visual comparison across panels. Pearson correlation coefficients (r) and Spearman rank correlation coefficients (ρ) with corresponding *p*-values are shown in the upper left corner of each panel. CRP, C-reactive protein; ICU, intensive care unit; WBC, white blood cell.

**Figure 2:**
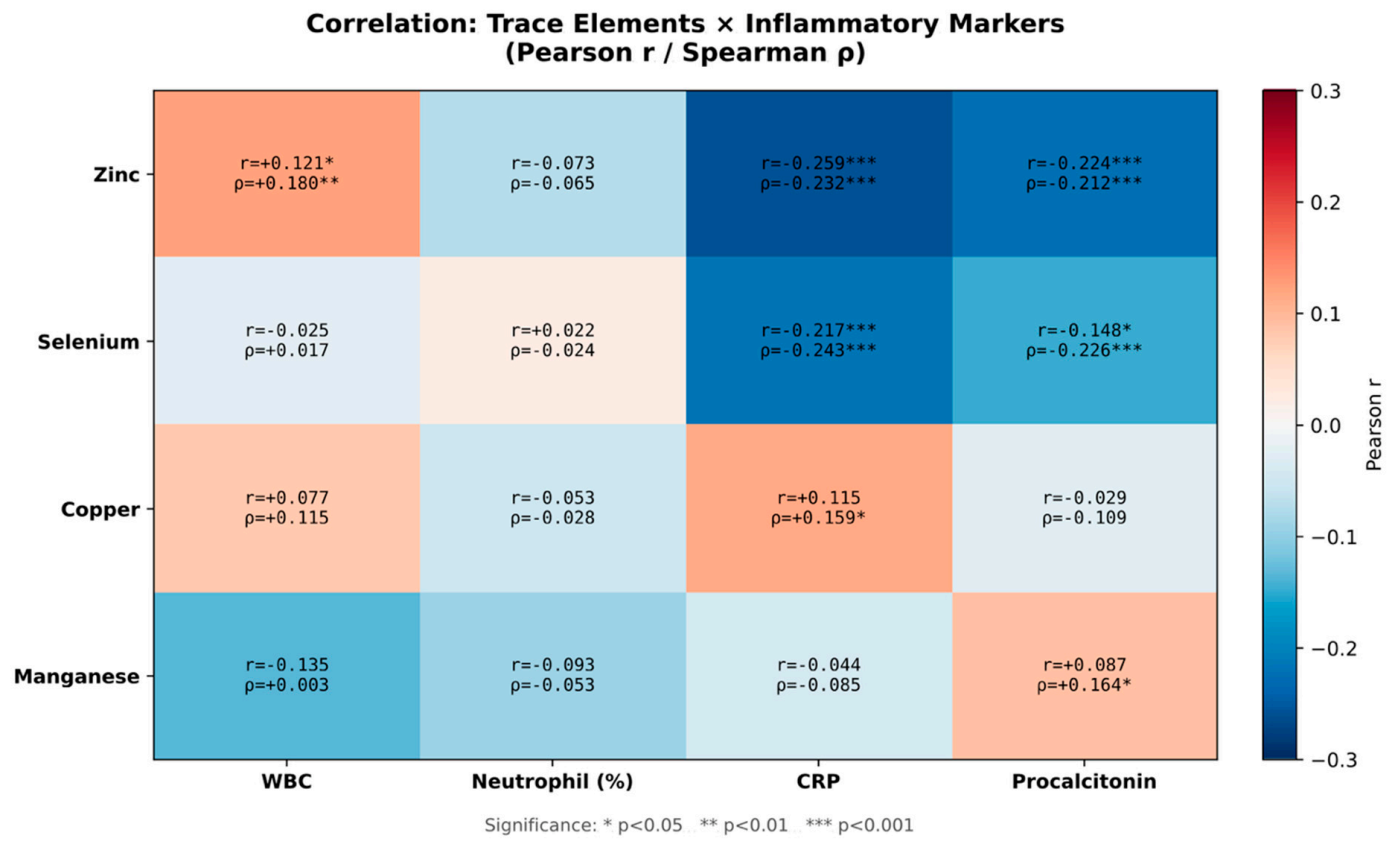
Heatmap of bivariate correlations between trace elements and inflammatory markers at ICU admission. Each cell displays the Pearson correlation coefficient (*r*; upper value) and Spearman rank correlation coefficient (ρ; lower value) for each trace element–inflammatory marker pair. Color intensity reflects the magnitude of the Pearson *r*, with red indicating positive and blue indicating negative correlations; the color scale ranges from −0.3 to +0.3. Asterisks denote statistical significance: * *p* < 0.05; ** *p* < 0.01; *** *p* < 0.001. CRP, C-reactive protein; ICU, intensive care unit; WBC, white blood cell.

The following analyses are based on trace element and inflammatory marker levels measured concurrently at ICU admission. Among the 16 trace element–marker combinations examined at ICU admission, statistically significant correlations were identified primarily for zinc and selenium with the acute-phase markers CRP and procalcitonin (Table 2, Figure 1 and Figure 2). Zinc showed significant negative correlations with CRP (Pearson r = −0.259, *p* < 0.001; Spearman ρ = −0.232, *p* < 0.001) and procalcitonin (r = −0.224, *p* < 0.001; ρ = −0.212, *p* < 0.001). Selenium was similarly negatively correlated with CRP (r = −0.217, *p* < 0.001; ρ = −0.243, *p* < 0.001) and procalcitonin (r = −0.148, *p* = 0.019; ρ = −0.226, *p* < 0.001). A borderline positive correlation was noted between zinc and WBC (r = 0.121, *p* = 0.050; ρ = 0.180, *p* = 0.003). No significant Pearson correlations were identified for copper or manganese, with the exception of a modest Spearman correlation between copper and CRP (ρ = 0.159, *p* = 0.044). No trace element correlated significantly with neutrophil percentage.

In an additional sex-stratified correlation analysis, the inverse associations of zinc and selenium with CRP were observed in both men and women. Zinc was also negatively associated with procalcitonin in both sex subgroups, whereas the selenium–procalcitonin association reached statistical significance in men but not in women. The main findings of this exploratory analysis are summarized in Supplementary Table S5.

### 3.3. Regression Analysis

**Figure 3:**
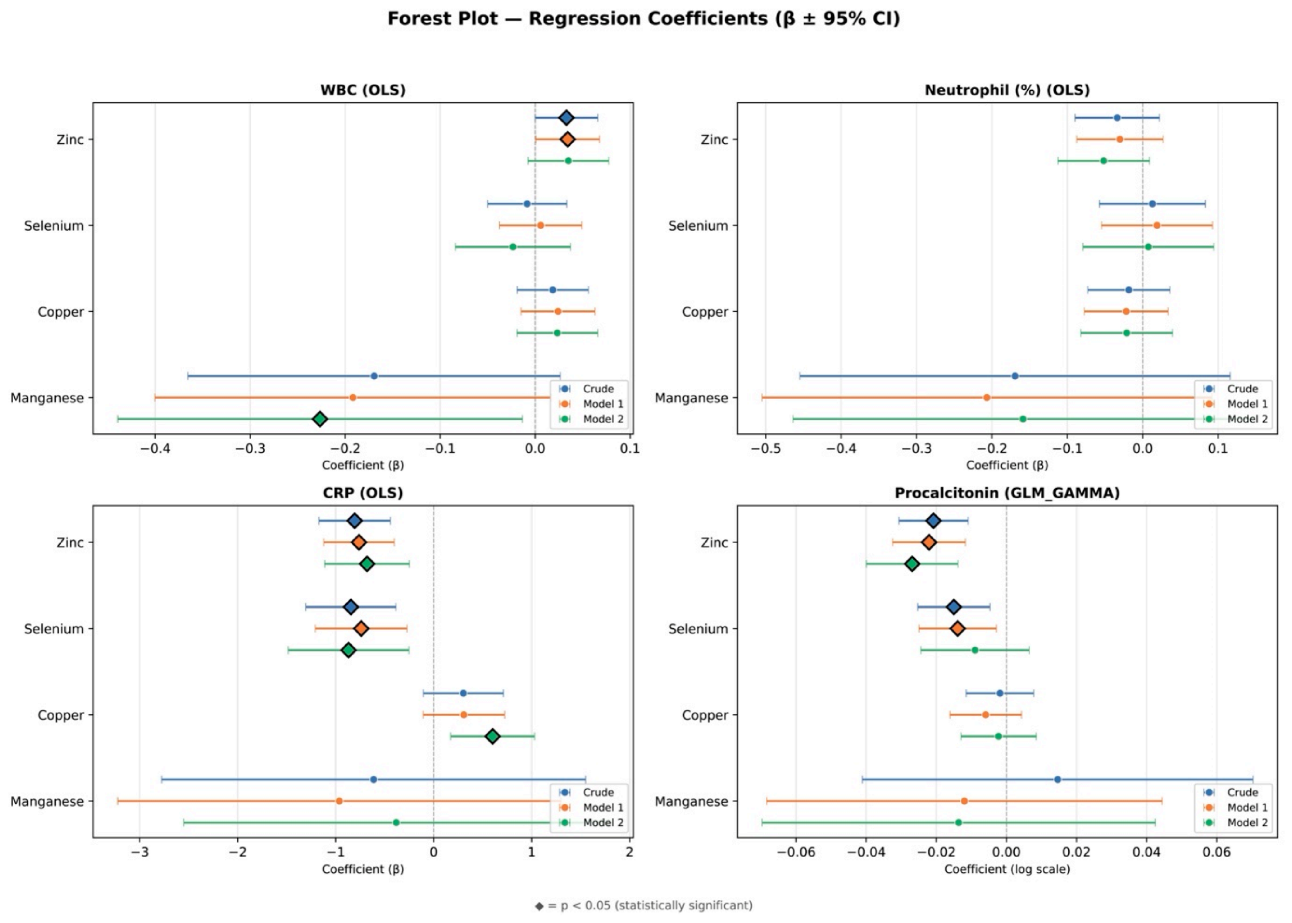
Forest plot of regression coefficients for the association between trace element levels at ICU admission and inflammatory markers. Each panel displays regression coefficients (β ± 95% CI) for the association of zinc, selenium, copper, and manganese with each inflammatory marker across three models: crude (blue), Model 1 (orange; adjusted for sex, age, APACHE II score, albumin, creatinine, and CRRT), and Model 2 (green; further adjusted for the remaining three trace elements). WBC, neutrophil percentage, and CRP were analyzed using ordinary least squares (OLS) regression; coefficients are expressed on the original scale. Procalcitonin was analyzed using a generalized linear model with Gamma distribution and log-link function; coefficients are expressed on the log scale. Filled diamonds (◆) indicate statistical significance (*p* < 0.05). The dashed vertical line represents the null value (β = 0). Horizontal lines represent 95% confidence intervals. APACHE, Acute Physiology and Chronic Health Evaluation; CI, confidence interval; CRP, C-reactive protein; CRRT, continuous renal replacement therapy; GLM, generalized linear model; ICU, intensive care unit; OLS, ordinary least squares; WBC, white blood cell.

Results of the hierarchical regression analyses are presented in Table 3, Table 4, Table 5 and Table 6 and Figure 3.

CRP. Both zinc (β = −0.81; 95% CI, −1.17 to −0.44; *p* < 0.001) and selenium (β = −0.84; 95% CI, −1.30 to −0.38; *p* < 0.001) showed significant negative associations with CRP in the crude model (Table 3). These associations were maintained after adjustment for clinical covariates in Model 1 (zinc: β = −0.76, *p* < 0.001; selenium: β = −0.74, *p* = 0.002) and persisted in the fully adjusted Model 2 (zinc: β = −0.68; 95% CI, −1.11 to −0.25; *p* = 0.002; selenium: β = −0.87; 95% CI, −1.48 to −0.25; *p* = 0.006). Copper showed a significant positive association with CRP exclusively in Model 2 (β = 0.60; 95% CI, 0.17 to 1.03; *p* = 0.006), a pattern consistent with suppression that was unmasked after mutual adjustment for other trace elements. Manganese was not significantly associated with CRP in any model.

Procalcitonin. Given its right-skewed distribution, GLM with Gamma family and log-link was applied (Table 4). Zinc was significantly associated with lower procalcitonin levels across all three models (crude: exp[β] = 0.979, *p* < 0.001; Model 1: exp[β] = 0.978, *p* < 0.001; Model 2: exp[β] = 0.973; 95% CI, 0.961–0.986; *p* < 0.001), corresponding to an approximately 2.7% decrease in expected procalcitonin per 1 µg/dL increase in zinc in the fully adjusted model. Selenium showed a significant association in the crude model (exp[β] = 0.985, *p* = 0.004) and Model 1 (*p* = 0.014) that was attenuated to non-significance in Model 2 (*p* = 0.257). Neither copper nor manganese was significantly associated with procalcitonin in any model.

WBC. Zinc showed a borderline positive association in the crude model (β = 0.033; 95% CI, 0.00–0.07; *p* = 0.050) and Model 1 (β = 0.034; *p* = 0.046), which was attenuated in Model 2 (*p* = 0.105; Table 5). Manganese was significantly and negatively associated with WBC only in Model 2 (β = −0.23; 95% CI, −0.44 to −0.01; *p* = 0.037).

Neutrophil percentage. No significant associations were observed between any trace element and neutrophil percentage across all three models (all *p* > 0.05; Table 6).

Because copper and manganese were available in a smaller proportion of the cohort, inclusion of these elements in Model 2 reduced the analyzable sample. We therefore performed a sensitivity analysis (Model 2a) in which zinc and selenium were mutually adjusted for one another, together with the same clinical covariates, but not for copper or manganese, thereby retaining nearly the full cohort. In this model, the association between zinc and CRP was essentially unchanged and estimated with greater precision (β = −0.70; 95% CI, −1.07 to −0.34; *p* < 0.001; n = 261), as was the association between zinc and procalcitonin (exp[β] = 0.979; 95% CI, 0.969 to 0.990; *p* < 0.001; n = 248). Selenium remained significantly associated with CRP (β = −0.59; 95% CI, −1.07 to −0.11; *p* = 0.016), although the point estimate was attenuated relative to the fully adjusted model, suggesting that adjustment for copper and manganese may influence the selenium estimate.

### 3.4. Serial-Level Analysis

To assess whether the associations observed at admission reflected a sustained pattern across the ICU stay, the analyses were repeated using per-patient median trace element levels derived from all available serial measurements. The serial-level results were broadly consistent with the initial-level findings.

Correlation analysis. Serial median zinc levels showed significant negative correlations with CRP (r = −0.261, *p* < 0.001; ρ = −0.243, *p* < 0.001) and procalcitonin (r = −0.223, *p* < 0.001; ρ = −0.199, *p* = 0.002), consistent with the initial-level findings, as well as a significant positive correlation with WBC (r = 0.128, *p* = 0.038; ρ = 0.169, *p* = 0.006). Selenium showed significant negative correlations with CRP (r = −0.235, *p* < 0.001; ρ = −0.280, *p* < 0.001) and procalcitonin (r = −0.181, *p* = 0.004; ρ = −0.282, *p* < 0.001), with magnitudes comparable to or modestly stronger than at admission. Full correlation results are presented in Supplementary Table S1 and Supplementary Figure S1.

Regression analysis. In the fully adjusted Model 2, the pattern of significant associations was largely consistent with the initial-level analysis (Supplementary Tables S2–S4 and Supplementary Figure S2). For CRP, zinc (β = −0.67; 95% CI, −1.09 to −0.25; *p* = 0.002) and selenium (β = −0.85; 95% CI, −1.43 to −0.27; *p* = 0.004) again demonstrated significant negative independent associations, while copper showed a significant positive association (β = +0.59; 95% CI, 0.19–0.99; *p* = 0.004; Supplementary Table S3). For procalcitonin, zinc retained a significant negative association in Model 2 (exp[β] = 0.975; corresponding to a 2.5% decrease per 1 µg/dL increase; 95% CI, −3.9% to −1.2%; *p* < 0.001), whereas selenium did not reach statistical significance (*p* = 0.067; Supplementary Table S4). In the exploratory WBC analysis, zinc reached significance in Model 2 (β = 0.057; 95% CI, 0.016–0.097; *p* = 0.006) and manganese retained a significant negative association (β = −0.23; 95% CI, −0.43 to −0.02; *p* = 0.031; Supplementary Table S2).

Taken together, the serial-level findings corroborate the initial-level results, indicating that the inverse associations of zinc and selenium with CRP, and of zinc with procalcitonin, are not confined to the admission time point but reflect a consistent pattern throughout the ICU stay.

## 4. Discussion

In this retrospective study of 267 critically ill ICU patients, we examined the independent associations between serum zinc, selenium, copper, and manganese concentrations and four concurrently measured inflammatory markers using hierarchical multivariable regression with three levels of adjustment. The principal finding was that lower zinc levels were independently and robustly associated with higher CRP and procalcitonin concentrations across all models and replicated in the serial-level analysis. Lower selenium independently predicted higher CRP in all models, while its association with procalcitonin was attenuated following full mutual adjustment. Copper showed a positive independent association with CRP exclusively in the fully adjusted model, and manganese showed a negative association with WBC in the serial-level analysis only.

The robust inverse associations between zinc and both CRP and procalcitonin, persisting through Crude, Model 1, and Model 2 and replicating in the serial-level analysis, represent the central and most consistent finding of this study. This is biologically plausible on multiple levels. At the molecular level, zinc exerts anti-inflammatory effects primarily through attenuation of NF-κB signaling: zinc deficiency augments NF-κB p65 DNA-binding activity in vivo, resulting in upregulated transcription of TNF-α, IL-1β, and IL-6 [6], while zinc repletion upregulates the NF-κB inhibitory protein A20, constraining cytokine-driven inflammation [7,14]. These mechanisms have been demonstrated in both experimental sepsis models [6] and in human supplementation trials, in which oral zinc supplementation in elderly subjects significantly reduced plasma CRP, IL-6, and macrophage chemotactic protein-1 compared to placebo [15]. In another randomized trial of healthy volunteers, zinc supplementation suppressed LPS-stimulated TNF-α and IL-1β mRNA expression in mononuclear cells and attenuated NF-κB activation ex vivo [14]. Mechanistically, TNF-α and IL-6 are the principal upstream drivers of the acute-phase response that culminates in hepatic CRP and procalcitonin synthesis, providing a direct link between zinc depletion and elevated inflammatory marker concentrations [16].

In the clinical context, the inverse relationship between zinc and CRP is further supported by cytokine-mediated hypozincemia: inflammatory signals, particularly IL-6, promote hepatic sequestration of zinc via upregulation of ZIP-family zinc transporters and metallothionein, simultaneously driving down serum zinc while amplifying acute-phase protein synthesis [5,9]. This bidirectional relationship—in which inflammation causes zinc redistribution while zinc deficiency amplifies the inflammatory response—represents a key interpretive challenge in observational studies such as ours [16]. The fact that the zinc–CRP association persisted in Model 2 after adjustment for albumin, illness severity (APACHE II), and co-existing trace elements is reassuring with respect to independent signal; however, causal directionality cannot be established from cross-sectional data. Notably, Suruli et al. [17] found that zinc deficiency was highly prevalent (86%) in critically ill patients but did not correlate with APACHE II or SOFA scores, suggesting that the zinc–inflammatory marker association in our data may not be purely a proxy for illness severity—a finding consistent with an independent modulatory role of zinc, although prospective confirmation is required.

The independent negative association of selenium with CRP, maintained through Model 2 after adjustment for clinical covariates and the other trace elements, parallels findings from prior studies. Ghashut et al. [5] demonstrated that plasma selenium was inversely correlated with CRP (ρ = −0.489) in a large nutritional cohort, with the relationship persisting after albumin adjustment. Selenium exerts anti-inflammatory effects through its incorporation into selenoproteins—particularly glutathione peroxidase isoforms and thioredoxin reductases—which neutralize lipid peroxides and reactive oxygen species that would otherwise sustain NF-κB-dependent cytokine transcription [8]. Early observational studies in critically ill patients demonstrated that plasma selenium concentrations inversely correlated with APACHE II and SAPS II scores, and that low plasma selenium at ICU admission was associated with three-fold higher rates of ventilator-associated pneumonia, organ failure, and mortality [18,19], establishing the biological and clinical relevance of selenium depletion in the ICU. These clinical associations are mechanistically coherent with our finding of an independent inverse selenium–CRP relationship.

The attenuation of the selenium–procalcitonin association in Model 2 is noteworthy. A prospective study in critically ill children found that plasma selenium on ICU admission was inversely associated with procalcitonin (β = −0.99; 95% CI, −1.64 to −0.34; *p* = 0.003) in unadjusted analysis [20], but the association diminished after adjustment for clinical severity. In our Model 2, the addition of zinc as a covariate—which shares overlapping inflammatory regulatory pathways—likely contributed to attenuation through multicollinearity. Additionally, selenium’s influence on procalcitonin may be more indirectly mediated through infection susceptibility rather than direct transcriptional regulation of the procalcitonin gene, whereas zinc acts more proximally on the NF-κB–cytokine–acute phase axis [6,8].

The significant positive association between copper and CRP emerging exclusively in Model 2 is a statistically instructive finding that reflects the known biology of ceruloplasmin, the principal copper-carrying protein in plasma. Ceruloplasmin is a well-characterized positive acute-phase reactant whose hepatic synthesis is upregulated in response to IL-6 signaling in parallel with CRP [2,3], and serum copper levels consequently tend to rise—rather than fall—during acute illness, a pattern documented in cohort studies of critically ill patients [4]. In unadjusted and Model 1 analyses, the positive copper–CRP relationship was suppressed by confounding from zinc and selenium, both of which co-vary inversely with copper in this population and simultaneously inversely with CRP. Removing this confounding by mutual adjustment in Model 2 unmasked the underlying positive association—a suppression effect consistent with the known acute-phase biology of ceruloplasmin [2,3]. This pattern underscores the importance of simultaneous multi-element analysis and cautions against interpreting single-element crude associations in isolation [3,16].

The negative association between manganese and WBC observed in the serial-level Model 2 only was not replicated in the initial-level analysis and should be interpreted with caution. Although manganese is an essential cofactor for antioxidant enzymes and theoretically could modulate leukocyte biology through oxidative stress pathways [2], the clinical significance of this finding is uncertain: the association emerged in only one of six models tested for this outcome, the confidence interval was wide, and no confirmatory prospective data exist in critically ill patients. This result warrants replication in larger studies before any mechanistic interpretation is offered.

The absence of significant associations between any trace element and neutrophil percentage across all analyses was not unexpected. Neutrophil percentage reflects the relative composition of the leukocyte differential and is primarily governed by corticosteroid exposure, demargination during physiological stress, bone marrow reserve, and infection source control—factors unlikely to be directly regulated by trace element concentrations [3]. This finding is consistent with prior systematic reviews in which no significant relationship between trace element status and neutrophil proportion was identified [3].

The serial-level analysis, in which per-patient medians of all available measurements during the ICU stay were used as summary exposures, largely replicated the admission-level results for the key associations: zinc with CRP (*p* = 0.002) and procalcitonin (*p* < 0.001), selenium with CRP (*p* = 0.004), and copper with CRP (*p* = 0.004). This consistency across two methodologically distinct analyses—cross-sectional admission measurements versus a summary exposure integrated across the ICU course—strengthens the inferential weight of these findings. The zinc–WBC association reaching statistical significance in the serial-level Model 2 (β = 0.057; *p* = 0.006) but not at admission likely reflects greater statistical power from the reduction in random measurement error achieved by averaging multiple time points, rather than a true admission-level effect.

Several methodological features support the robustness of the findings. The simultaneous assessment of four trace elements with concurrent hierarchical regression, including mutual adjustment in Model 2, allowed decomposition of the independent contribution of each element while controlling for co-element confounding—an approach rarely applied in prior ICU trace element studies, which typically analyze elements in isolation [12,13]. The supplementary serial-level analysis provided an independent internal replication with a summary exposure measure, lending longitudinal plausibility to the primary findings. Concurrent measurement of trace elements and inflammatory markers at the same time points reduced ascertainment bias inherent in temporally misaligned assessments.

Several limitations require careful consideration. First and most critically, the retrospective, observational design precludes causal inference. The inverse associations observed between zinc or selenium and inflammatory markers are consistent with a potential immunomodulatory role, but they are equally consistent with epiphenomenal hypozincemia and hyposelenemia driven by the inflammatory process itself [16]. Interventional studies are needed to establish directionality. Second, while albumin was included as a covariate in Model 1, trace element concentrations were not albumin-corrected; given the documented positive association between albumin and both zinc and selenium, residual confounding by hypoalbuminemia cannot be excluded [5]. Third, copper and manganese were measured in only a subset of the cohort, and these data were not missing completely at random. The pattern of missingness was informative: missingness for copper and manganese was almost entirely concordant, and nearly all patients fell into one of two measurement patterns—either all four trace elements or zinc and selenium alone. This suggests that missingness arose at the level of the laboratory panel ordered by the treating physician rather than from random failure of individual assays, consistent with the retrospective design in which trace element testing was performed according to clinical indication rather than a uniform protocol. To assess whether this missingness was likely to bias our estimates, we compared patients in whom copper and manganese were measured with those in whom they were not. The two groups did not differ significantly in age, sex, APACHE II score, albumin, creatinine, or CRRT use. Importantly, they also did not differ in any of the four inflammatory outcomes: WBC count, neutrophil percentage, CRP, or procalcitonin. Significant differences were confined to serum zinc and in-hospital mortality. We therefore cannot assume that these data were missing completely at random. However, because missingness was not associated with the inflammatory outcomes under study, complete-case estimates are less likely to be substantially biased by outcome-dependent missingness. Nonetheless, the reduced effective sample size for models including copper and manganese limits precision, and residual bias arising from unmeasured determinants of testing cannot be excluded. Fourth, the inclusion of copper and manganese as covariates in the fully adjusted model reduced the analyzable sample and consequently the precision of those estimates. To address this, we conducted a sensitivity analysis restricted to mutual adjustment between zinc and selenium, which retained nearly the entire cohort. The associations of zinc with CRP and procalcitonin were essentially unchanged and were estimated with narrower confidence intervals, indicating that the principal findings were not an artefact of reduced sample size. The selenium–CRP association likewise remained significant, although its magnitude was attenuated. Estimates for copper and manganese themselves remain less precise and should be regarded as provisional. Fifth, several biologically plausible confounders were not available in our dataset. Information on glucocorticoid and antibiotic administration, the route and composition of nutritional support—including elemental supplementation—and CRRT filtration dose was not systematically retrievable from the retrospective records; CRRT was captured only as a binary variable. This is particularly relevant with respect to nutritional support, as parenteral and enteral formulations may contain trace elements and therefore directly influence serum trace element concentrations. Glucocorticoids and antibiotics may independently alter leukocyte kinetics and acute-phase protein synthesis, and the intensity of renal replacement therapy may influence trace element clearance. Residual confounding from these unmeasured factors cannot be excluded. Sixth, serial measurements were obtained based on clinical indication rather than a standardized protocol, making it impossible to distinguish patient-driven variation from random measurement timing. Seventh, as a single-center study of a predominantly elderly, high-acuity Korean ICU population (median APACHE II score, 29; in-hospital mortality, 44.2%), generalizability to other clinical settings with different case-mix, nutritional practices, or trace element supplementation protocols may be limited.

The consistent independent associations between zinc and CRP and procalcitonin—observed across admission and serial levels and maintained after multiple covariate adjustments—support a role for routine trace element monitoring in critically ill patients. Evidence from randomized trials suggests that combined antioxidant supplementation, including selenium, in septic ICU patients reduces oxidative stress and infectious complications [21], and that early enteral pharmaconutrition containing zinc and selenium improves organ function recovery compared to standard care [22]. These findings provide a translational rationale for prospective investigation of whether targeted trace element repletion in hypozincemic or hyposelenemic ICU patients can attenuate systemic inflammatory burden and improve clinical outcomes. The copper–CRP suppression effect further highlights the importance of interpreting individual trace element concentrations in the context of the full multi-element profile and concurrent inflammatory state rather than in isolation.

## 5. Conclusions

In this retrospective study of critically ill ICU patients, serum zinc and selenium concentrations were independently and inversely associated with acute-phase inflammatory markers—most consistently, zinc with both CRP and procalcitonin, and selenium with CRP—after adjustment for clinical covariates and co-existing trace element levels. These associations were not confined to the admission time point but were also observed in per-patient serial median analyses throughout the ICU stay, suggesting that they reflected a sustained pattern rather than a single time-point finding. Copper demonstrated a positive independent association with CRP in the fully adjusted model, consistent with its role as a component of the positive acute-phase reactant ceruloplasmin.

Collectively, these findings support the potential clinical relevance of interpreting trace element status in conjunction with systemic inflammation and the broader multi-element profile in critically ill patients. However, given the retrospective observational design and the possibility of residual confounding, these results should not be interpreted as evidence that trace element depletion causes inflammation or that trace element repletion directly attenuates inflammatory burden. Prospective studies with standardized trace element measurement and detailed documentation of nutritional support, medication exposure, and renal replacement therapy parameters are warranted to determine whether targeted correction of trace element deficiencies can modify inflammatory trajectories or improve clinical outcomes.

## Figures and Tables

**Table 1:** Baseline Characteristics of the Study Population (*N* = 267).

Variable	Overall (*N* = 267)	Missing, *n*
Demographics		
Age, years	72.0 (60.5–80.0)	
Male sex	170 (63.7)	
Clinical parameters		
APACHE II score	29.0 (20.0–35.0)	2
Albumin, g/dL	2.7 (2.5–3.0)	
Creatinine, mg/dL	0.9 (0.6–1.6)	
CRRT use	49 (18.4)	
In-hospital mortality	118 (44.2)	
Trace elements		
Zinc, µg/dL	49.0 (37.0–66.0)	1
Selenium, µg/L	54.0 (44.0–66.8)	1
Copper, µg/dL	75.0 (58.2–94.8)	105
Manganese, µg/L	1.3 (0.9–1.9)	106
Inflammatory markers		
WBC, ×103/µL	11.3 (7.3–15.4)	2
Neutrophil, %	86.1 (78.9–90.6)	2
CRP, mg/L	126.3 (54.4–181.1)	
Procalcitonin, ng/mL	2.3 (0.6–10.7)	13

Values are median (IQR) for continuous variables and *n* (%) for categorical variables. APACHE, Acute Physiology and Chronic Health Evaluation; CRP, C-reactive protein; CRRT, continuous renal replacement therapy; IQR, interquartile range; WBC, white blood cell.

**Table 2:** Bivariate Correlations Between Trace Elements and Inflammatory Markers.

Trace Element	WBC	Neutrophil (%)	CRP	Procalcitonin
Zinc	r = +0.121 * ρ = +0.180 *	r = −0.073 ρ = −0.065	r = −0.259 *** ρ = −0.232 ***	r = −0.224 *** ρ = −0.212 ***
Selenium	r = −0.025 ρ = +0.017	r = +0.022 ρ = −0.024	r = −0.217 *** ρ = −0.243 ***	r = −0.148 * ρ = −0.226 ***
Copper	r = +0.077 ρ = +0.115	r = −0.053 ρ = −0.028	r = +0.115 ρ = +0.159 *	r = −0.029 ρ = −0.109
Manganese	r = −0.135 ρ = +0.003	r = −0.093 ρ = −0.053	r = −0.044 ρ = −0.085	r = +0.087 ρ = +0.164 *

r = Pearson correlation coefficient; ρ = Spearman rank correlation coefficient. Bold values indicate statistical significance. Significance: * *p* < 0.05; ** *p* < 0.01; *** *p* < 0.001. CRP, C-reactive protein; WBC, white blood cell.

**Table 3:** Hierarchical Regression Analysis for CRP (OLS).

Exposure	Crude (*N* = 266)		Model 1 (*N* = 264)		Model 2 (*N* = 160)	
	β (95% CI)	*p*	β (95% CI)	*p*	β (95% CI)	*p*
Zinc	**−0.81** **(−1.17 to −0.44)**	**<0.001**	**−0.76** **(−1.12 to −0.40)**	**<0.001**	**−0.68** **(−1.11 to −0.25)**	**0.002**
Selenium	**−0.84** **(−1.30 to −0.38)**	**<0.001**	**−0.74** **(−1.21 to −0.27)**	**0.002**	**−0.87** **(−1.48 to −0.25)**	**0.006**
Copper	0.30 (−0.11 to 0.71)	0.146	0.31 (−0.11 to 0.72)	0.146	**0.60** **(0.17 to 1.03)**	**0.006**
Manganese	−0.61 (−2.78 to 1.55)	0.577	−0.96 (−3.22 to 1.30)	0.401	−0.38 (−2.55 to 1.79)	0.728

Crude: univariate (trace element only). Model 1: adjusted for sex, age, APACHE II score, albumin, creatinine, and CRRT. Model 2: further adjusted for the remaining three trace elements. Bold values indicate statistical significance (*p* < 0.05). β = unstandardized regression coefficient (OLS). APACHE, Acute Physiology and Chronic Health Evaluation; CRP, C-reactive protein; CRRT, continuous renal replacement therapy.

**Table 4:** Hierarchical Regression Analysis for Procalcitonin (GLM, Gamma Distribution, Log-Link).

Exposure	Crude (*N* = 253)		Model 1 (*N* = 251)		Model 2 (*N* = 151)	
	exp(β) [95% CI]	*p*	exp(β) [95% CI]	*p*	exp(β) [95% CI]	*p*
Zinc	**0.979** **(0.970 to 0.989)**	**<0.001**	**0.978** **(0.968 to 0.988)**	**<0.001**	**0.973** **(0.961 to 0.986)**	**<0.001**
Selenium	**0.985** **(0.975 to 0.995)**	**0.004**	**0.986** **(0.975 to 0.997)**	**0.014**	**0.991** **(0.976 to 1.007)**	**0.257**
Copper	0.998 (0.989 to 1.008)	0.711	0.994 (0.984 to 1.004)	0.257	**0.998** **(0.987 to 1.009)**	**0.684**
Manganese	1.015 (0.960 to 1.073)	0.606	0.988 (0.934 to 1.045)	0.677	0.986 (0.933 to 1.043)	0.634

Crude: univariate (trace element only). Model 1: adjusted for sex, age, APACHE II score, albumin, creatinine, and CRRT. Model 2: further adjusted for the remaining three trace elements. Bold values indicate statistical significance (*p* < 0.05). exp(β) represents the multiplicative change in the expected procalcitonin per unit increase in the trace element. APACHE, Acute Physiology and Chronic Health Evaluation; CRRT, continuous renal replacement therapy; GLM, generalized linear model.

**Table 5:** Hierarchical Regression Analysis for WBC (OLS).

Exposure	Crude (*N* = 264)		Model 1 (*N* = 262)		Model 2 (*N* = 159)	
	β (95% CI)	*p*	β (95% CI)	*p*	β (95% CI)	*p*
Zinc	**0.033** **(0.000 to 0.067)**	**0.050**	**0.034** **(0.001 to 0.068)**	**0.046**	0.033 (−0.007 to 0.073)	0.105
Selenium	−0.014 (−0.053 to 0.026)	0.690	0.005 (−0.037 to 0.047)	0.792	−0.022 (−0.079 to 0.036)	0.448
Copper	0.016 (−0.016 to 0.049)	0.329	0.021 (−0.012 to 0.054)	0.222	0.021 (−0.016 to 0.058)	0.278
Manganese	−0.170 (−0.368 to 0.028)	0.090	−0.187 (−0.393 to 0.018)	0.071	**−0.226** **(−0.438 to −0.013)**	**0.037**

Crude: univariate (trace element only). Model 1: adjusted for sex, age, APACHE II score, albumin, creatinine, and CRRT. Model 2: further adjusted for the remaining three trace elements. Bold values indicate statistical significance (*p* < 0.05). β = unstandardized regression coefficient (OLS). APACHE, Acute Physiology and Chronic Health Evaluation; CRRT, continuous renal replacement therapy; WBC, white blood cell.

**Table 6:** Hierarchical Regression Analysis for Neutrophil Percentage (OLS).

Exposure	Crude (*N* = 264)		Model 1 (*N* = 262)		Model 2 (*N* = 159)	
	β (95% CI)	*p*	β (95% CI)	*p*	β (95% CI)	*p*
Zinc	−0.034 (−0.090 to 0.022)	0.234	−0.030 (−0.087 to 0.027)	0.300	−0.047 (−0.103 to 0.010)	0.095
Selenium	0.015 (−0.055 to 0.084)	0.719	0.022 (−0.046 to 0.090)	0.612	0.006 (−0.073 to 0.086)	0.864
Copper	−0.019 (−0.074 to 0.036)	0.507	−0.022 (−0.079 to 0.034)	0.437	−0.019 (−0.078 to 0.041)	0.492
Manganese	−0.165 (−0.451 to 0.120)	0.243	−0.205 (−0.499 to 0.088)	0.173	−0.157 (−0.456 to 0.143)	0.305

Crude: univariate (trace element only). Model 1: adjusted for sex, age, APACHE II score, albumin, creatinine, and CRRT. Model 2: further adjusted for the remaining three trace elements. Bold values indicate statistical significance (*p* < 0.05). β = unstandardized regression coefficient (OLS). APACHE, Acute Physiology and Chronic Health Evaluation; CRRT, continuous renal replacement therapy.

## Data Availability

The data presented in this study are available on request from the corresponding author due to privacy restrictions.

## Supplementary Materials

The following supporting information can be downloaded at: https://www.mdpi.com/article/10.3390/nu18152508/s1, Figure S1: Correlation heatmap of trace elements and inflammatory markers based on serial median measurements; Figure S2: Forest plot of regression coefficients for the association between serial median trace element levels and inflammatory markers; Table S1: Bivariate correlations between trace elements and inflammatory markers (Serial Median); Table S2: Hierarchical regression analysis for WBC using serial median trace element levels (OLS); Table S3: Hierarchical regression analysis for CRP using serial median trace element levels (OLS); Table S4: Hierarchical regression analysis for procalcitonin using serial median trace element levels (GLM, Gamma Distribution, Log-Link); Table S5: Sex-stratified correlations between serum trace elements and inflammatory markers at ICU admission.
