# Supplementary material for: Serum Zinc and Selenium Are Independently Associated with Acute-Phase Inflammatory Markers in Critically Ill Patients: A Retrospective Observational Study
Source: Nutrients. 2026 Aug 3;18(15):2508. doi: 10.3390/nu18152508 (PMC13467620; doi:10.3390/nu18152508)
Supplement: Supplementary file 1 [file nutrients-18-02508-s001.zip › Supplementary_Table_S1-5.pdf]

Supplementary Table S1. Bivariate Correlations Between Trace Elements and Inflammatory Markers (Serial Median)

Trace Element	WBC	Neutrophil (%)	CRP	Procalcitonin
Zinc	**r = +0.128***<br>***ρ = +0.169******	r = −0.100<br>*ρ = −0.148**	**r = −0.261*******<br>***ρ = −0.243*******	**r = −0.223*******<br>***ρ = −0.199******
Selenium	r = −0.019<br>*ρ = +0.040*	r = +0.028<br>*ρ = −0.067*	**r = −0.235*******<br>***ρ = −0.280*******	**r = −0.181******<br>***ρ = −0.282*******
Copper	r = +0.021<br>*ρ = +0.037*	r = −0.046<br>*ρ = −0.085*	r = +0.114<br>*ρ = +0.142*	r = −0.041<br>*ρ = −0.109*
Manganese	r = −0.124<br>*ρ = +0.086*	r = −0.060<br>*ρ = −0.013*	r = −0.012<br>*ρ = −0.015*	r = +0.101<br>*ρ = +0.175**

Significance: * p < 0.05; ** p < 0.01; *** p < 0.001.
r = Pearson correlation coefficient; ρ = Spearman rank correlation coefficient.
Bold values indicate statistical significance (*p* < 0.05).
Trace element exposures represent the per-patient median of all available serial measurements obtained during the ICU stay. CRP, C-reactive protein; ICU, intensive care unit; WBC, white blood cell.

Supplementary Table S2. Hierarchical Regression Analysis for WBC Using Serial Median Trace Element Levels (OLS)

Exposure	Crude (N = 264)		Model 1 (N = 262)		Model 2 (N = 172)	
β (95% CI)	*p*	*β (95% CI)*	*p*	*β (95% CI)*	*p*	
Zinc	**0.033 (0.002 to 0.063)**	**0.038**	**0.033 (0.002 to 0.065)**	**0.038**	**0.057 (0.016 to 0.097)**	**0.006**
Selenium	−0.006 (−0.045 to 0.033)	0.761	0.006 (−0.035 to 0.047)	0.777	−0.023 (−0.078 to 0.033)	0.426
Copper	0.005 (−0.030 to 0.040)	0.780	0.010 (−0.027 to 0.047)	0.589	0.003 (−0.036 to 0.042)	0.878
Manganese	−0.158 (−0.349 to 0.033)	0.104	−0.165 (−0.367 to 0.037)	0.108	**−0.226 (−0.430 to −0.022)**	**0.031**

Crude: univariate (trace element only). Model 1: additionally adjusted for sex, age, APACHE II score, albumin, creatinine, and CRRT. Model 2: further adjusted for the remaining three trace elements. Bold values indicate statistical significance ($p < 0.05$).

β = unstandardized regression coefficient (OLS). APACHE, Acute Physiology and Chronic Health Evaluation; CRRT, continuous renal replacement therapy; WBC, white blood cell.

Supplementary Table S3. Hierarchical Regression Analysis for CRP Using Serial Median Trace Element Levels (OLS)

**Exposure**	**Crude (N = 266)**		**Model 1 (N = 264)**		**Model 2 (N = 174)**	
β (95% CI)	***p***	***β (95% CI)***	***p***	***β (95% CI)***	***p***	
Zinc	**−0.758 (−1.098 to −0.417)**	**<0.001**	**−0.766 (−1.103 to −0.428)**	**<0.001**	**−0.672 (−1.089 to −0.255)**	**0.002**
Selenium	**−0.851 (−1.278 to −0.424)**	**<0.001**	**−0.810 (−1.251 to −0.368)**	**<0.001**	**−0.852 (−1.430 to −0.275)**	**0.004**
Copper	0.296 (−0.087 to 0.678)	0.129	0.325 (−0.067 to 0.716)	0.103	**0.593 (0.192 to 0.993)**	**0.004**
Manganese	−0.169 (−2.300 to 1.962)	0.876	−0.738 (−2.962 to 1.486)	0.513	−0.260 (−2.380 to 1.861)	0.809

Crude: univariate (trace element only). Model 1: additionally adjusted for sex, age, APACHE II score, albumin, creatinine, and CRRT. Model 2: further adjusted for the remaining three trace elements. Bold values indicate statistical significance ($p < 0.05$).

β = unstandardized regression coefficient (OLS). APACHE, Acute Physiology and Chronic Health Evaluation; CRP, C-reactive protein; CRRT, continuous renal replacement therapy.

Supplementary Table S4. Hierarchical Regression Analysis for Procalcitonin Using Serial Median Trace Element Levels (GLM, Gamma Distribution, Log-Link)

**Exposure**	**Crude (N = 253)**		**Model 1 (N = 251)**		**Model 2 (N = 164)**	
β (95% CI) / exp(β)	***p***	***β (95% CI) / exp(β)***	***p***	***β (95% CI) / exp(β)***	***p***	
Zinc	**−0.0195 (−0.0298 to −0.0092) exp(β) = 0.981**	**<0.001**	**−0.0245 (−0.0347 to −0.0144) exp(β) = 0.976**	**<0.001**	**−0.0256 (−0.0391 to −0.0120) exp(β) = 0.975**	**<0.001**
Selenium	**−0.0203 (−0.0313 to −0.0093) exp(β) = 0.980**	**<0.001**	**−0.0217 (−0.0339 to −0.0094) exp(β) = 0.979**	**<0.001**	−0.0145 (−0.0301 to 0.0010) exp(β) = 0.986	0.067
Copper	−0.0029 (−0.0126 to 0.0069) exp(β) = 0.997	0.564	−0.0056 (−0.0159 to 0.0047) exp(β) = 0.994	0.284	−0.0026 (−0.0134 to 0.0082) exp(β) = 0.997	0.636
Manganese	0.0175 (−0.0413 to 0.0763) exp(β) = 1.018	0.559	−0.0107 (−0.0688 to 0.0474) exp(β) = 0.989	0.719	−0.0160 (−0.0750 to 0.0430) exp(β) = 0.984	0.595

Crude: univariate (trace element only). Model 1: additionally adjusted for sex, age, APACHE II score, albumin, creatinine, and CRRT. Model 2: further adjusted for the remaining three trace elements. Bold values indicate statistical significance ($p < 0.05$).

β = regression coefficient on the log scale. exp(β) represents the multiplicative change in expected procalcitonin per 1-unit increase in the trace element. APACHE, Acute Physiology and Chronic Health Evaluation; CRRT, continuous renal replacement therapy; GLM, generalized linear model.

Supplementary Table S5. Sex-stratified correlations between serum trace elements and inflammatory markers at ICU admission

Association	Male	Female
Zinc-CRP	r = −0.258, p < 0.001	r = −0.316, p < 0.01
Zinc-Procalcitonin	r = −0.222, p < 0.01	r = −0.240, p < 0.05
Selenium-CRP	r = −0.209, p < 0.01	r = −0.329, p < 0.01
Selenium-Procalcitonin	r = −0.180, p < 0.05	r = −0.127, NS
Zinc-WBC	ρ = 0.211, p < 0.01	r = 0.187, NS
Manganese-Procalcitonin	NS	ρ = 0.292, p < 0.05

Values are shown as Pearson correlation coefficients (r) or Spearman rank correlation coefficients (ρ), as appropriate. Only associations with at least borderline or statistically significant findings in either sex subgroup are shown. CRP, C-reactive protein; NS, not significant; WBC, white blood cell.
